# Higher Imported Food Patterns Are Associated with Obesity and Severe Obesity in Tuvalu: A Latent Class Analysis

**DOI:** 10.1016/j.cdnut.2024.102080

**Published:** 2024-01-24

**Authors:** José Francisco López-Gil, Stephanie M Wu, Tai-Lin (Irene) Lee, Chih-Wei Shih, Selotia Tausi, Vine Sosene, Pauke P Maani, Malo Tupulaga, Yu-Tien Hsu, Chia-Rui Chang, Shi-Chian Shiau, Yuan-Hung Lo, Chih-Fu Wei, Po-Jen Lin, Maria Soledad Hershey

**Affiliations:** 1 One Health Research Group, Universidad de Las Américas, Quito, Ecuador; 2 Department of Biostatistics, Harvard T.H. Chan School of Public Health, Boston, MA, United States; 3 Department of Radiology and Imaging Science, Emory University School of Medicine, Atlanta, GA, United States; 4 Taiwan International Cooperation and Development Fund (ICDF), Taipei, Taiwan; 5 Taiwan Technical Mission to Tuvalu, Funafuti, Tuvalu; 6 Ministry of Local Government and Agriculture, Department of Agriculture, Tuvalu; 7 Department of Public Health, Ministry of Health, Tuvalu; 8 Department of Social & Behavioral Sciences, Harvard T.H. Chan School of Public Health, Boston, MA, United States; 9 Department of Environmental Health, Harvard T.H. Chan School of Public Health, Boston, MA, United States; 10 Bloomberg School of Public Health, Johns Hopkins University, Baltimore, MD, United States; 11 Department of Medicine, Nuvance Health Danbury Hospital, Danbury, CT, United States

**Keywords:** global health, public health nutrition, climate, food systems, diet, nutrition and health

## Abstract

**Background:**

Tuvalu is a Pacific Island country within the small island developing states that has observed a significant and alarming increase in obesity rates over the past 40 years, affecting ∼60 %−70 % of the current population.

**Objectives:**

This study aimed to investigate the association between food patterns and the proportion of obesity in a Pacific Island country.

**Methods:**

The 2022 COMmunity-based Behavior and Attitude survey in Tuvalu (COMBAT) included 985 adults with complete data on sociodemographic information and the frequency of consumption of 25 common foods. A latent class analysis determined 4 food patterns. Bayesian multilevel logistic and linear regression models estimated the association between food patterns and the proportion of obesity [body mass index (BMI) ≥30 kg/m^2^], severe obesity (BMI ≥40 kg/m^2^), and weight (kg), adjusting for potential confounders and accounting for clustering by region.

**Results:**

The latent class analysis revealed 4 food patterns with an entropy of 0.94 and an average posterior probability of class assignment for each individual of 0.97, described as follows: *1*) local: locally produced foods with moderate food diversity (proportion of individuals = 28 %); *2*) diverse-local: local with greater food diversity (17 %); *3*) restricted-imported: more imported with restricted diversity (29 %); and *4*) imported: heavily imported with high diversity (26 %). Compared to those following the diverse-local pattern, the odds of having obesity were greater for those classified with the imported pattern [odds ratio (OR): 2.52; 95 % credible interval (CrI): 1.59, 3.99], restricted-imported pattern (OR: 1.89; 95 % CrI: 1.59, 3.99), and local pattern (OR: 1.54; 95 % CrI: 0.94, 2.50). Similar trends were observed for severe obesity while body weight was positively associated with both restricted-imported and imported food patterns.

**Conclusions:**

The high consumption of imported foods, together with the low consumption of plant-based foods and protein-rich foods, could be a relevant modifiable lifestyle factor explaining the high levels of obesity and severe obesity in Tuvalu, a Pacific Island country.

## Introduction

The global prevalence of obesity (i.e., excess weight) poses a substantial hurdle to promoting lifelong health and effectively preventing chronic diseases such as but not limited to stroke, coronary artery disease, hypertension, type 2 diabetes, dyslipidemia, and cancer [1,2]. The global prevalence of obesity has increased almost threefold since 1975 [3]. In 2016, >650 million adults aged 18 years and older were affected by obesity [3]. Over the past few decades, Pacific Island nations in particular have suffered a rapid increase in obesity and noncommunicable diseases, including type 2 diabetes, cancer, cardiovascular diseases [4]. Specifically, in Tuvalu, a Pacific Island country within the small island developing states, there has been a significant and alarming increase in obesity rates over the past 40 years [3,5,6]. The burden of obesity is extremely high in Tuvalu individuals, and obesity affects ∼60 %−70 % of the population. According to the World Health Organization STEPwise approach to noncommunicable disease risk factor surveillance (STEPS) Country Report in Tuvalu (2015), >60 % of the population between the ages of 18 and 69 was found to have obesity [7]. Additionally, recent data from the Global Nutrition Report in 2021 revealed a high prevalence of obesity in Tuvalu, with 59.9 % of adult females and 51.5 % of adult males being affected [8].

Although obesity has become a worldwide epidemic, there is limited agreement regarding its underlying causes [9]. Obesity is a complex disease characterized by excessive adiposity that can impair health [10] and has a complex etiology [11]; this disease encompasses the interplay of biological, social, environmental, and genetic factors, among other variables [12]. Regarding environmental factors, the World Health Organization has identified 2 prominent factors that play a major role in the escalating prevalence of obesity: *1*) a substantial increase in the consumption of high-calorie foods with high sugar and fat content and *2*) increasingly sedentary behavior driven by changes in work patterns, transportation choices, and urbanization [2]. Consequently, lifestyle modifications, which include changing dietary habits to reduce the overall intake of energy, in addition to other factors, appear to be crucial for effectively managing body weight with long-term sustainability [13].

For the inhabitants of Tuvalu, globalization has greatly changed their way of life, mainly due to a continuous shift in eating habits from healthy, nutrient-rich foods to the consumption of imported staples and highly processed food products [14]. Moreover, Tuvalu faces the triple threat of obesity, climate change, and food insecurity. Composed of 9 small coral islands (∼12,000 inhabitants), Tuvalu has little arable land. This issue has been exacerbated by soil salinization, which has led to subsequent decreases in domestic vegetable and fruit supplies. As a result, Tuvaluans now import most of their food. These imported foods are usually high in salt, fat, and sugar and often lack essential nutrients [15]. Consequently, more efforts have been made at the local, regional, and international levels to increase the importance of nutrition in the country. The high proportion of obesity in the Tuvalu population is likely intertwined with these factors, and further research is needed to identify achievable, sustainable lifestyle changes and culturally sensitive interventions. However, to our knowledge, no studies have evaluated the association of dietary patterns of Tuvalu with obesity.

For Tuvalu to achieve a 25 % reduction in premature mortality from noncommunicable diseases by 2025 [7], a better understanding of the current food consumption patterns and potential associations with obesity-related outcomes is warranted. In this sense, a latent class analysis (LCA) using a model-based clustering approach can serve as an effective tool to analyze the consumption patterns of a full set of foods collected in a comprehensive diet assessment [16]. Therefore, the aims of this study were twofold: *1*) to determine the different patterns of food consumption among the Tuvaluan population using LCA and *2*) to examine the associations of the derived dietary patterns with weight and the odds of having obesity and severe obesity among people in this Pacific Island country. It was hypothesized that an imported food pattern would be associated with a higher probability of having obesity among our adult study population compared to those with a diverse consumption of culturally conventional foods.

## Methods

### Study population

The COMmunity-based Behavior and Attitude survey in Tuvalu (COMBAT) collected data between February and May 2022 from 1030 adults (985 with complete data) from Funafuti, the main island, and the other 8 outlying islands [17]. In Funafuti, interviewers visited households in the 7 villages on Fongafale Islets (Fakaifou, Senala, Alapi, Vaiaku, Lofeagai, Teone, and Tekavatoetoe). On the outlying islands, interviewers used passenger ships to visit households around the island wharf, the primary residential area. A convenience sample selection process was used to enroll native and immigrant Tuvaluan citizens aged 18 y or older, allowing 1 or 2 study participants (aged >18 y) to come from each household. Due to missing information, 45 adults (4 %) were not included in the analysis, with no systematic missingness detected using Little’s missing completely at random test [18]. The study protocol was approved by the Tuvalu Department of Health and more specifications on the study design and population have been published previously [17].

### Measures

#### Dietary patterns (independent variable)

Questionnaires administered in person by trained interviewers in Tuvaluan were used to capture individual-level sociodemographic information and the consumption frequency of 25 common food items: rice, potatoes, swamp taro or taro, cassava, breadfruit, instant noodles, fish, chicken, pork, lamb or beef, cabbage, cucumber, bird’s nest fern, imported vegetables, banana, papaya, coconut, pandanus, imported fruits, milk, eggs, chips or biscuits, sweetened beverages, cakes, and ice cream. The list of food items was generated after discussion with Taiwan International Cooperation and Development Fund staff and local coordinators from the Tuvalu Department of Health to determine food items that were most common and would best reflect the dietary habits of the Tuvaluans. Each food item response had 5 categories of consumption frequency: almost every day, several times a week, several times a month, less than once a month, and never. To improve interpretability and minimize sparsity, these data were recoded into 3 categories: *1*) everyday/more than once weekly, *2*) more than once per month, and *3*) less than once a month/never.

#### Anthropometric information (dependent variable)

Anthropometric data were collected by locally trained staff who measured participants’ height with a tape measure and weight with an electronic weight during in-person interviews. BMI was calculated as weight (kg) divided by height (m) squared. Obesity in adults was defined by a BMI ≥30 kg/m^2^, whereas obesity class III or severe obesity was defined as a BMI ≥40 kg/m^2^.

#### Covariates

The interview questionnaire was structured to collect information regarding home garden use, sociodemographic information, health behaviors and attitudes, and self-reported medical history. Covariates included age (centered around the mean age of the study population), education level (high school education or higher compared with less than high school), smoking status (smoker compared with nonsmoker), exercise level (every day, several times a week, or several times a month), and history of noncommunicable disease (NCD) (self-reported hypertension, diabetes, or dyslipidemia compared with none). We further accounted for geographical clustering (Funafuti compared with other islands) in our Bayesian multilevel model.

### Statistical analysis

We conducted an LCA [19] using all 25 food items to identify food patterns in the population. Four food patterns were identified, and individuals were classified into the corresponding 4 latent classes. The number of latent classes was obtained by using maximum likelihood estimation to fit models with an increasing number of classes. Then, the model with the best fit was determined based on discussions with local nutrition experts, ensuring class sizes were above 5 % [20], and elbow plots of statistical criteria, including the Bayesian information criteria [21], the consistent Akaike information criteria [22], and the log-likelihood [23], were examined. Diagnostic criteria to ensure proper separation of classes included an entropy value [24] of 0.94 and an average latent class posterior probability [25] of 0.97, both of which satisfied the recommended cutoff of 0.8 [20]. Individuals were assigned to 1 of the 4 latent classes based on which class had the highest posterior probability. The distribution of sociodemographic variables by latent class was then examined to understand the heterogeneity of food pattern adoption across Tuvalu.

To estimate the association between food patterns and the outcomes of obesity, severe obesity, and weight, we included latent class as a covariate and separately modeled each outcome using a Bayesian multilevel regression model, adjusting for age, gender, physical activity, smoking status, chronic disease prevalence, and education level, with random effects accounting for clustering by region. Logistic regression models were conducted for obesity and severe obesity; moreover, a linear regression model was used for weight, with standard weakly informative priors [26]. Posterior parameter estimates and equal-tailed 95 % posterior credible intervals were reported for all outcomes. For obesity and severe obesity, the posterior prevalence for each latent class was calculated using the odds for the reference level and the odds ratio (OR) for the remaining levels. We also reported the posterior error probability to control for multiple comparisons [27]. Stratified analyses for obesity were conducted based on sex, region, education, smoking status, chronic disease prevalence, and exercise level.

To conduct all the analyses, we used R version 4.2.1 [28]. The “poLCA” package version 1.6.0.1 [29] was used for the LCA, the “naniar” package version 1.0.0 [30] was used for Little’s missing completely at random test, and the “rstanarm” package version 2.21.3 [31] was used for the Bayesian logistic and linear regression analyses. For each model, 4 chains of 2000 iterations were implemented with a target average acceptance probability of 0.999. Convergence was assessed by the effective sample size and potential scale reduction factors for all parameters [32].

## Results

### Description of food patterns

**FIGURE 1:**
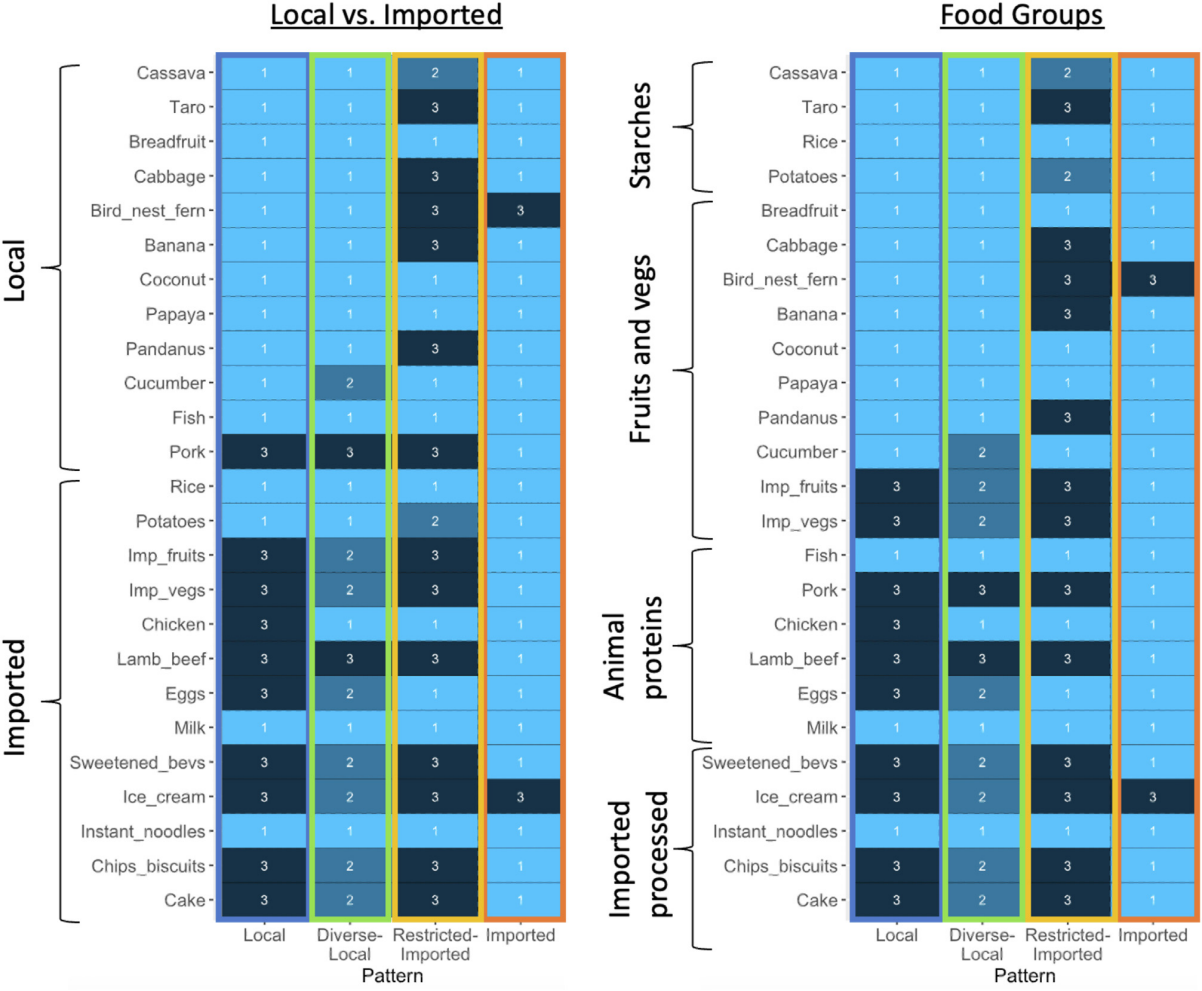
Modal consumption levels for the 4 latent food patterns derived from the Tuvalu COMBAT survey, classified by local compared with imported food production (left) and by food groups (right). The COMmunity-based Behavior and Attitude survey in Tuvalu (COMBAT survey) collected the frequency of consumption of 25 common food items. Each food item response had 5 categories that were recoded into 3 levels: everyday/more than once weekly (level 1), more than once per month (level 2), and less than once per month/never (level 3). Modal consumption represents the highest probability for the frequency of consumption of each food group among participants classified by dietary pattern.

Four distinct food patterns were identified based on the self-reported intake frequency among 985 adult participants from the 9 islands of Tuvalu. These patterns were local (27.7 %), diverse-local (17.3 %), restricted-imported (29.3 %), and imported (25.7 %). An average latent class posterior probability of 0.97 indicated a high level of confidence in this classification. The local pattern featured a higher frequency of domestic food products, such as taro, bird nest fern, and coconut, with relatively lower intake of animal proteins. The diverse-local pattern exhibited a mixed intake of both local and imported food products, with greater food diversity compared to the local pattern, particularly for animal proteins, fruits, and vegetables. The restricted-imported pattern displayed the most restricted food diversity and was characterized by imported items such as rice and instant noodles accompanied by local items such as breadfruit, coconut, and fish. Finally, participants classified with the imported food pattern showed the greatest overall food diversity, with frequent consumption of all the foods, except bird nest fern and ice cream, and displayed the highest consumption of imported food items among the 4 derived patterns (Figure 1).

### Sociodemographic characteristics of the study population

The participants were mainly from Funafuti (60.6 %), the main island in Tuvalu, and there were slightly more females (53.7 %) than males in the study population. Most of the participants were middle aged (median age: 38 y; interquartile range [IQR]: 28–55), whereas few had completed a high school education or higher. There were 228 (23.2 %) participants who reported a history of hypertension, diabetes, or dyslipidemia (summarized as NCD), and 334 participants smoked. Finally, 24.2 % of the study population reported an exercise frequency less than once per week. Among the participants in the COMBAT study population in 2022, 73 % had obesity, and 22 % had severe obesity.

The distribution of sociodemographic variables according to the 4 food intake patterns revealed that the proportion of residents on Tuvalu’s outlying islands was high among populations following the diverse-local (46.5 %) and local (90.8 %) patterns, and the ages of the local food pattern were higher (median: 52 y; IQR: 34–61). Although there was a higher percentage of self-reported NCD (37.7 %) in the local food pattern, a greater percentage of current smokers were observed in the diverse-local food pattern (50.6 %). Infrequent or low exercise levels were observed in 37.8 % of the restricted-imported food pattern users. Moreover, the distributions of males and females were similar across all 4 food patterns (Table 1).

**TABLE 1:** Sociodemographic characteristics by food patterns in the COMmunity-based Behavior and Attitude survey in Tuvalu (COMBAT) population

Sociodemographic characteristics	Food patterns				
	Overall	Diverse-local	Local	Restricted-imported	Imported
*N*	985	170	273	289	253
Residence outside of main island (%)	388 (39.4)	79 (46.5)	248 (90.8)	11 (3.8)	50 (19.8)
Female (%)	529 (53.7)	88 (51.8)	145 (53.1)	151 (52.2)	145 (57.3)
Age, median y (IQR)	38.0 (28.0–55.0)	34.5 (29.0–47.0)	52.0 (34.0–61.0)	32.0 (27.0–43.0)	40.0 (26.8–55.0)
High school education or greater (%)	151 (15.3)	22 (12.9)	21 (7.7)	45 (15.6)	63 (24.9)
Self-reported NCD^1^ (%)	228 (23.2)	28 (16.6)	103 (37.7)	34 (11.8)	63 (25.0)
Current smoker; yes vs. no (%)	334 (33.9)	86 (50.6)	92 (33.7)	86 (29.8)	70 (27.8)
Exercise level^2^ (%)					
High	331 (33.8)	54 (31.8)	102 (37.6)	77 (26.7)	98 (39.0)
Medium	412 (42.0)	83 (48.8)	130 (48.0)	102 (35.4)	97 (38.6)
Low	237 (24.2)	33 (19.4)	39 (14.4)	109 (37.8)	56 (22.3)
BMI, median kg/m^2^ (IQR)	33.8 (29.6–38.8)	31.1 (28.3–36.3)	34.6 (30.5–40.1)	33.1 (29.1–38.8)	35.0 (30.9–40.6)

Abbreviations: BMI, body mass index; IQR, interquartile range, NCD, noncommunicable disease.

Column-wise percentages in parentheses can be interpreted as the percentage of participants with the sociodemographic characteristic among those who follow the given food pattern or the entire study population (“Overall”).

1 NCD was defined as having hypertension, diabetes, or dyslipidemia in the study population.

2 Exercise level was recorded as low: less than once a week, medium: several times a week, high: every day.

### Associations between food patterns and the incidence of obesity, severe obesity, and body weight

The posterior prevalence and odds of obesity (BMI ≥30 kg/m^2^), severe obesity (BMI ≥40 kg/m^2^), and posterior mean weight are shown in Table 2, and the full regression model results are shown in Supplemental Table 1. The posterior prevalence of obesity and severe obesity were lowest for those with a diverse-local food intake pattern (0.59 and 0.07, respectively); the posterior mean weight for the diverse-local food pattern group was 92.28 kg [95 % credible interval (CrI): 77.53, 110.46].

**TABLE 2:** Associations between the derived food patterns and the prevalence of obesity, severe obesity, and weight in Tuvalu 2022

Food patterns	Obesity (BMI ≥30 kg/m^2^)				Severe obesity (BMI ≥40 kg/m^2^)				Weight (kg)		
	Post Prev	Post OR	95 % Cred Int	PEP	Post Prev	Post OR	95 % Cred Int	PEP	Post mean difference	95 % Cred Int	PEP
Diverse-local (Ref.)	0.59	1	NA	NA	0.07	1	NA	NA	92.28	(77.53, 110.46)	0
Local	0.69	1.57	(0.94, 2.56)	0.04	0.13	2.09	(1.20, 3.66)	0.004	2.18	(−2.04, 6.49)	0.166
Restricted-imported	0.73	1.91	(1.19, 3.02)	0.002	0.12	1.79	(1.03, 3.17)	0.02	5.13	(1.14, 9.16)	0.009
Imported	0.78	2.54	(1.61, 4.09)	<0.001	0.17	2.70	(1.55, 4.76)	<0.001	9.40	(5.42, 13.44)	<0.001

Abbreviations: Cred Int, credible interval; PEP, posterior error probability for multiple comparisons; NA, not applicable; Post OR, posterior odds ratio; Post Prev, posterior prevalence; Ref., reference.

The multivariable models were adjusted for sex, age, education, smoking status, and physical activity level, with random effects accounting for clustering by region.

PEP: *P* (odds ratio ≤ 1) is the posterior error probability that the true odds ratio is in the opposite direction from the observed estimate. This can be interpreted as a local false discovery rate and provides information to help control multiple comparisons.

The posterior prevalence was calculated based on the posterior odds for the reference level (not displayed) and the odds ratios for the remaining levels.

The imported food pattern had significantly greater posterior odds for obesity (OR, 2.54; 95 % CrI: 1.61, 4.09) and severe obesity (OR: 2.70; 95 % CrI: 1.55, 4.76) than did the diverse-local food pattern after we adjusted for potential confounders. Similar associations were also observed for the restricted-imported food pattern (obesity OR: 1.91; 95 % CrI: 1.19, 3.02; severe obesity OR: 1.79; 95 % CrI: 1.03, 3.17). The local pattern was associated with greater odds of severe obesity (OR: 2.09; 95 % CrI: 1.20, 3.66) but not obesity (OR: 1.57; 95 % CrI: 0.94, 2.56). Direct associations with body weight were also found among consumers of the Restricted-imported [posterior weight difference: 5.13; 95 % CrI: 1.14, 9.16; posterior error probability (PEP): 0.009] and imported food patterns (posterior weight difference: 9.40; 95 % CrI: 5.42, 13.44; PEP: <0.001) groups compared to diverse-local consumers, supporting the findings for obesity. Posterior error probabilities showed consistent results with the 95 % CrI after accounting for multiple comparisons by controlling for the local false discovery rate. Similar trends were observed when fitting the marginal models without adjusting for additional covariates (Supplemental Table 2).

### Stratified analysis by different demographic factors

Table 3 shows that the association between the imported food pattern and obesity was significantly stronger in individuals with less than high school education (OR: 1.93; 95 % CrI: 1.11, 3.46), nonsmokers (OR: 2.16; 95 % CrI: 1.08, 4.43), those without self-reported NCD diagnosis (OR: 1.93; 95 % CrI: 1.03, 3.69), and individuals with medium exercise levels (OR: 4.35; 95 % CrI: 1.62, 12.36). Moreover, the Local pattern was associated with lower odds of having obesity among females (OR: 0.49; 95 % CrI: 0.24, 0.99*)* and among current smokers (OR: 0.38; 95 % CrI: 0.18, 0.79).

**TABLE 3:** Subgroup analyses of the association between the derived food patterns and the incidence of obesity (BMI ≥30 kg/m^2^) across sociodemographic characteristics

			Food patterns			
Subgroups	*N*	Obesity cases	Diverse-local	Local	Restricted-imported	Imported
			Posterior OR (95 % credible interval)			
*Sex*						
Male	522	385	1 (Ref.)	0.75 (0.39, 1.47)	1.09 (0.54, 2.38)	1.32 (0.67, 2.68)
Female	449	323	1 (Ref.)	0.49 (0.24, 0.99)^†^	1.16 (0.55, 2.77)	2.03 (0.95, 4.73)
*Region*						
Main island	585	412	1 (Ref.)	0.69 (0.25, 1.86)	1.50 (0.56, 3.76)	2.00 (0.75, 5.21)
Outlying islands	386	296	1 (Ref.)	0.69 (0.39, 1.22)	1.40 (0.30, 9.98)	1.16 (0.54, 2.63)
*Education*						
Less than high school	824	598	1 (Ref.)	0.65 (0.40, 1.06)	1.17 (0.67, 2.13)	1.93 (1.11, 3.46)^†^
High school or greater	147	110	1 (Ref.)	0.21 (0.03, 1.68)	0.76 (0.09, 6.86)	0.48 (0.06, 3.43)
*Smoking status*						
Nonsmokers	642	481	1 (Ref.)	0.85 (0.45, 1.67)	1.48 (0.74, 3.11)	2.16 (1.08, 4.43)^†^
Current smokers	329	227	1 (Ref.)	0.38 (0.18, 0.79*)*^†^	0.78 (0.33, 2.02)	0.93 (0.41, 2.17)
*NCD*						
None	745	532	1 (Ref.)	0.59 (0.34, 1.05)	1.55 (0.80, 2.94)	1.93 (1.03, 3.69)^†^
Self-reported NCD^1^	226	176	1 (Ref.)	1.63 (0.50, 7.07)	0.43 (0.12, 1.40)	0.92 (0.32, 2.62)
*Exercise level*^2^						
High	329	229	1 (Ref.)	0.57 (0.27, 1.20)	1.12 (0.46, 2.82)	1.16 (0.55, 2.72)
Medium	407	311	1 (Ref.)	0.83 (0.36, 1.91)	1.73 (0.65, 4.89)	4.35 (1.62, 12.36)^†^
Low	235	168	1 (Ref.)	0.45 (0.13, 1.41)	0.67 (0.22, 2.00)	0.65 (0.22, 2.04)

Abbreviations: NCD, noncommunicable disease; OR, odds ratio; Ref., reference.

Multivariate models were adjusted for sex, age, education, smoking status, and physical activity level, excluding the corresponding stratification variable, with random effects accounting for clustering by region.

^†^ indicates a *p*-value less than 0.05.

1 NCD was defined as having hypertension, diabetes, or dyslipidemia in the study population.

2 Exercise level was recorded as low: less than once a week, medium: several times a week, high: every day.

## Discussion

### Main findings

To date, this is the first study to assess the relationship between food patterns and obesity in Tuvalu. Overall, we observed 4 different food consumption patterns among the Tuvalu populations, which were identified as local, diverse-local, restricted-imported, and imported. Compared to participants with the diverse-local pattern, participants with the restricted-imported or imported pattern had greater odds of having obesity or severe obesity, as well as higher mean body weight. Moreover, the local pattern was associated with severe obesity. The strongest associations, in terms of the likelihood of having obesity or severe obesity, were found for participants with an imported food pattern after we adjusted for several sociodemographic and lifestyle covariates. Our results, based on the limited available evidence on Pacific Island dietary habits, support the association between food patterns and obesity-related outcomes. These findings reinforce the notion that such countries could benefit from nutrition monitoring systems to fully understand changing diets and inform effective policy interventions [33]. In view of the alarming prevalence of obesity in Tuvalu [8], these findings could be useful for developing healthy intervention strategies to reduce the burden of obesity in this country, such as importing more nutritious and less processed foods, encouraging home garden cultivation of traditional local foods, and encouraging other healthy diet and lifestyle habits, discussed in more detail below. Although these eating patterns are understood as a sum of their components [34], differences in some individual food groups, the locality of food production, and the food diversity that differentiates the 4 observed eating patterns could explain these findings in part.

### The role of ultra-processed foods in obesity

Our results indicated that participants with a food pattern characterized by a high consumption of imported foods (e.g., sugar-sweetened foods, ice cream, chips, biscuits, cake) had the highest odds of having obesity and severe obesity compared with those with a diverse-local food pattern. One possible reason could be related to the greater degree of processing of imported foods [35]. The small island developing states in the Pacific (e.g., Tuvalu) heavily depend on imported food items that are frequently calorie dense but nutrient poor and tend to be rich in salt, fat, and sugar [15]. The consumption of ultra-processed foods has been associated with a higher risk of noncommunicable diseases, including excess weight [36]. Similarly, a greater consumption of ultra-processed foods is associated with a greater incidence of overweight and obesity [[37], [38], [39]]. Moreover, ultra-processed foods (e.g., sugar-sweetened beverages, chips, biscuits) are typically consumed at a significantly faster rate than locally sourced foods, which are less processed and often consist of nonimported ingredients (e.g., fruits, vegetables, starches). Consequently, the faster consumption of ultra-processed foods may also contribute to greater caloric intake [40]. Ultra-processed foods also tend to be perceived as more palatable than unprocessed foods, which can impact the regulation of normal appetite [41]. This increased palatability may result in increased caloric intake and contribute to the development of obesity. However, because the avoidance of ultra-processed foods may also cause possible adverse effects (e.g., reduced diet quality, increased risk of food poisoning and food wastage) [42], these hypotheses should be interpreted with caution and warrant future studies to verify causality and plausible mechanisms of the deleterious health effects of ultra-processed foods. However, because we do not know the exact degree of processing of all imported foods, this hypothesis should be interpreted with caution.

### The role of plant-based foods in individuals with obesity

Our results showed that participants with a restricted-imported food pattern, which included a lower consumption of fruits, vegetables, and starches, had greater odds of having obesity and severe obesity. This result is in line with a previous systematic review and meta-analysis that concluded that adherence to a plant-based diet is associated with lower body adiposity with a moderate level of certainty [43]. Similarly, the findings of one systematic review and dose‒response meta-analysis indicated that high intakes of fruits and vegetables (among others) were linked to a reduced risk of certain obesity-related conditions (e.g., overweight/obesity, abdominal obesity, weight gain) [44]. One possible explanation for this result could be the high volume of fruits and vegetables. High-volume foods require more time to consume than low-volume foods, extending the duration of a meal, which can enhance feelings of satiety and reduce overall energy intake [45]. Furthermore, when compared on an equal carbohydrate basis, meals containing potatoes (which are less consumed in those with a restricted-imported food pattern) have been found to be more satiating than meals containing rice or pasta, suggesting that potatoes are a more favorable option as a low-energy-dense food that can contribute to satiety [46]. Moreover, not only the volume but also the increase in fiber intake associated with fruit and vegetable consumption may be related to reducing risk of obesity and NCD [47].

### The role of protein-rich foods in individuals with obesity

Another possible explanation for our findings could lie in the role of protein intake. Although participants who adhered to the local pattern had high fish consumption (a common feature in all derived dietary patterns), they reported lower intakes of other protein-rich foods (i.e., pork, chicken, lamb, beef, and eggs) than participants with a diverse-local pattern. Protein is a macronutrient that provides high satiety power, meaning that it helps reduce appetite and increase feelings of fullness after meals. For instance, when comparing higher and lower protein intake levels (both below 1.6 g/kg/d), there is a substantial body of evidence consistently suggesting that higher protein diets have appetite-suppressing effects compared to lower protein diets [48]. Similarly, previous research has indicated that protein is the most satiating macronutrient in comparison with carbohydrates and fats [49] and may further stimulate the release of hormones that lower blood sugar levels and suppress appetite [50,51]. Moreover, protein intake has been linked to decreased energy intake, improved energy efficiency, and increased thermogenesis, all of which play a role in managing body weight [52].

### The role of sociodemographic and lifestyle variables in obesity

Although we controlled for sociodemographic factors such as age, sex, education level, and region, as well as lifestyle factors such as smoking and physical activity, it is worth noting the specific traits associated with the identified food consumption patterns. The proportion of participants with low physical activity levels (i.e., less than once a week) was greater for the restricted-imported and imported food patterns than for the diverse-local and local food patterns. Engaging in regular physical activity has been associated with various positive health outcomes, including lower levels of adiposity (i.e., body weight, body fat percentage, obesity) [53]. The lower level of participants engaging in low physical activity could be a possible explanation for this result. Although the analyses were adjusted for physical activity, those who exercised less may also be more likely to engage in other behaviors that are risk factors for obesity but were not measured in the COMBAT survey (i.e., optimal sleep duration, sedentary behaviors) [54].

Furthermore, the diverse-local food pattern had a greater proportion of individuals living outside of the main island (i.e., Funafuti) than did the Imported-restricted and Imported food patterns. People living in rural areas or outlying islands of Tuvalu may have limited access to processed foods. This may be due to a lack of commercial outlets or less importation of these products. In contrast, the incorporation of traditional local foods into the diet may contribute toward a higher diet quality. These foods tend to provide an enhanced intake of essential vitamins and minerals, dietary fiber, plant-based protein, and healthy fats [55,56], which could be related to a lower proportion of obesity and severe obesity. In addition, we observed that the highest proportion of participants with a high school education or higher were classified into the imported food pattern. The association between education level and obesity has been shown to be influenced by the specific measure of obesity (i.e., BMI, waist circumference) [57], sex, and economic development level of the country. An inverse association between education level and obesity is more common in higher-income countries (particularly for females) [57], whereas a positive association is more prevalent in lower-income countries; however, the nature of this association is inconsistent across studies and, therefore, should be interpreted with caution [58].

### Methodological considerations

This study has several limitations. First, due to the nature of a cross-sectional study, we are not able to establish causal relationships. Therefore, additional studies with longitudinal designs are warranted. Second, residual confounding factors may be present in the associations between the derived patterns and obesity or severe obesity (e.g., dietary practices, energy intake, sleep duration, sedentary behavior) that were not collected in the COMBAT survey. Nevertheless, we considered various potential sociodemographic and lifestyle factors in our multivariable models that could influence the associations between food group consumption patterns and obesity or severe obesity. Third, self-report questionnaires may introduce social desirability and recall bias. Fourth, effects may be slightly attenuated due to possible misclassification bias because regressions were performed successively without fully accounting for the measurement error in the classification steps. However, the results provide a lower bound on the associations of interest, so significant results remain informative and motivate confirmatory analyses in future research. Moreover, because the separation of classes was high, the attenuation effect was expected to be small. Fifth, although participants from the same households were included in some cases, this information was not reported in the survey. Thus, we were unable to account for the presence of correlated data. Last, we were not able to obtain some health-related information because the survey did not ask about specific information (e.g., pregnant or lactating mothers).

However, the main strength of this study is that we included a large sample of participants from all 9 islands that comprise the entire country of Tuvalu, which represents ∼10 % of this understudied population. However, given that a convenience sampling method was utilized, the generalizability of our findings is limited. Another strength is that we applied a model-based clustering approach (i.e., LCA) as an effective tool to analyze the consumption patterns of a full set of foods included in a locally tailored diet assessment comprehensively [16]. Finally, the COMBAT survey, which was designed in collaboration with local nutritionists, agricultural specialists, physicians, and epidemiologists, examined diet-related behavioral patterns and prevalent health outcomes across Tuvalu.

## Conclusions

The high consumption of imported foods, together with the low consumption of plant-based foods and protein-rich foods, could be a relevant modifiable lifestyle factor explaining the high levels of obesity and severe obesity in Tuvalu, a Pacific Island country. These results should be deemed clinically meaningful, given the prevalence of unhealthy eating patterns derived from self-reported habitual food consumption, as well as the high prevalence of obesity and severe obesity. Furthermore, these findings could have significant implications for public health, as they suggest that enhancing diet quality could serve as a preventive measure against obesity and severe obesity during the early stages of life. Nevertheless, future studies with larger sample sizes and longitudinal designs are warranted to further study this hypothesis and evaluate the medium- and long-term impacts of diet quality among Tuvaluans, providing insight for future nutritional and food system strategies to combat obesity.

## Author contributions

The authors’ responsibilities were as follows – JFL-G, MSH, C-FW, P-JL, T-LL, SMW: designed the research; T-LL, C-WS, ST, VS, PPM, MT, Y-TH, C-RC, S-CS, Y-HL, C-FW, P-JL: conducted the research; SMW: analyzed the data; JFL-G, SMW, P-JL: wrote the article; MSH: had primary responsibility for the final content; and all authors: read and approved the final manuscript.

### Conflict of interest

P-JL and C-FW received consulting fees from the Taiwan Technical Mission in Tuvalu. P-JL received reimbursement from the Taiwan Technical Mission in Tuvalu for attending the Planetary Health Annual Meeting 2022 in Boston, MD, United States. All other authors report no conflicts of interest.

## Funding

The survey was funded by a collaborative effort between the Taiwan International Cooperation and Development Fund and the Tuvalu government as part of the Fruit and Vegetable Production and Nutrition Enhancement Project. Since 2011, the Tuvalu Department of Health and the Tuvalu Agriculture Department has been collaborating with the Taiwan Technical Mission to promote the development of family and government gardens, as well as providing health education and cooking sessions, to increase the yield of fruits and vegetables in Tuvalu and promote a healthier diet. MSH received ERC training-grant support (T42 OH008416). SMW is supported by the National Institute of Allergy and Infectious Diseases (NIAID: T32 AI007358).

## Data availability

The data described in the manuscript, code book, and analytic code will be made publicly and freely available without restriction on GitHub at https://github.com/smwu/tuvalu.

## Appendix A Supplementary data

Multimedia component1 Multimedia component1
