# Supplementary material for: Higher Imported Food Patterns Are Associated with Obesity and Severe Obesity in Tuvalu: A Latent Class Analysis
Source: Curr Dev Nutr. 2024 Jan 24;8(2):102080. doi: 10.1016/j.cdnut.2024.102080 (PMC10862406; doi:10.1016/j.cdnut.2024.102080)
Supplement: Multimedia component1 [file mmc1.docx]

**Supplemental Table 1**: Full regression model results for the association of the derived food patterns and covariates with the prevalence of obesity, morbid obesity, and weight in Tuvalu 2022.

|  | **Obesity (BMI ≥ 30kg/m^2^)** | | **Severe obesity (BMI ≥ 40kg/m^2^)** | | **Weight (kg)** | |
| --- | --- | --- | --- | --- | --- | --- |
|  | Post Odds or OR | 95% Cred Int | Post Odds or OR | 95% Cred Int | Post Mean difference | 95% Cred Int |
| Intercept | 1.41 | (0.33, 4.66) | 0.07 | (0.03, 0.25) | 92.28 | (77.53, 110.46) |
| **Dietary patterns** |  |  |  |  |  |  |
| Diverse-local (Ref) | 1 |  | 1 |  | 1 |  |
| Local | 1.57 | (0.94, 2.56) | 2.09 | (1.20, 3.66) | 2.18 | (-2.04, 6.49) |
| Restricted-imported | 1.91 | (1.19, 3.02) | 1.79 | (1.03, 3.17) | 5.13 | (1.14, 9.16) |
| Imported | 2.54 | (1.61, 4.09) | 2.70 | (1.55, 4.76) | 9.40 | (5.42, 13.44) |
| **Sex**; female | 1.00 | (0.74, 1.37) | 1.41 | (1.02, 1.93) | -1.93 | (-4.61, 0.78) |
| **Age** (Centered) | 1.02 | (1.01, 1.03) | 1.01 | (0.99, 1.02) | 0.07 | (-0.02, 0.16) |
| **College or greater** | 1.30 | (0.84, 2.06) | 1.09 | (0.68, 1.70) | 1.75 | (-1.96, 5.37) |
| **Current smoker** | 0.87 | (0.63, 1.20) | 1.12 | (0.79, 1.58) | 0.10 | (-2.92, 2.89) |
| **Exercise level**  Medium | 1.44 | (0.99, 2.01) | 1.89 | (1.30, 2.79) | 4.48 | (1.43, 7.57) |
| Low | 1.14 | (0.76, 1.73) | 1.47 | (0.95, 2.31) | 0.89 | (-2.71, 4.42) |
| **Self-reported NCD** | 0.97 | (0.64, 1.44) | 1.35 | (0.91, 1.99) | 2.05 | (-1.34, 5.42) |
| **Height** (m) (Centered) | N/A | N/A | N/A | N/A | 0.36 | (0.23, 0.48) |

Cred Int: credible intervals, N/A: not applicable, OR: odds ratio, m:meters, Ref: reference

Multivariable models are adjusted for sex, age, education, smoking status, and physical activity level, with random effects accounting for clustering by region.

*Intercept for age and height were set at mean value.

**Supplemental Table 2:** Marginal model results of the associations between the derived food patterns and the prevalence of obesity, severe obesity, and weight in Tuvalu 2022.

|  | **Obesity (BMI ≥ 30 kg/m^2^)** | | | | **Severe Obesity (BMI ≥ 40 kg/m^2^)** | | | | **Weight (kg)** | | |
| --- | --- | --- | --- | --- | --- | --- | --- | --- | --- | --- | --- |
| **Food patterns** | Post Prev | Post OR | 95% Cred Int | PEP | Post Prev | Post OR | 95% Cred Int | PEP | Post Mean difference | 95% Cred Int | PEP |
| Diverse-local (Ref.) | 0.615 | 1 | NA | NA | 0.131 | 1 | NA | NA | 95.251 | [80.488,113.070] | 0 |
| Local | 0.738 | 1.762 | [1.114, 2.775] | 0.009 | 0.249 | 2.195 | [1.298, 3.815] | 0.002 | 0.865 | [ -3.269, 4.982] | 0.344 |
| Restricted-imported | 0.735 | 1.734 | [1.104, 2.657] | 0.005 | 0.193 | 1.589 | [0.931, 2.824] | 0.049 | 2.984 | [ -0.921, 7.270] | 0.062 |
| Imported | 0.803 | 2.554 | [1.635, 4.046] | 0 | 0.28 | 2.578 | [1.550, 4.414] | 0 | 8.098 | [ 4.302, 12.269] | 0 |

PEP: posterior error probabilities for multiple comparisons, Post Prev: posterior prevalence, Post OR: posterior odds ratio, Cred Int: credible intervals, NA: not applicable, Ref.: reference.

Marginal models are unadjusted and random effects account for clustering by region to present crude estimates.

PEP: P(OR <= 1) is the posterior error probability that the true odds ratio is in the opposite direction from the observed estimate. It can be interpreted as a local false discovery rate and provides information to help control multiple comparisons.

Posterior prevalence is calculated based on the posterior odds for the reference level (not displayed) and the odds ratios for the remaining levels.
